# A family with Danon disease caused by a splice site mutation in *LAMP2* that generates a truncated protein

**DOI:** 10.1002/mgg3.561

**Published:** 2019-02-03

**Authors:** Nianwei Zhou, Jie Cui, Weipeng Zhao, Yingying Jiang, Wenqing Zhu, Lu Tang, Xuejie Li, Minmin Sun, Cuizhen Pan, Xianhong Shu

**Affiliations:** ^1^ Department of Echocardiography Zhongshan Hospital Shanghai Institute of Cardiovascular Disease Shanghai Institute of Medical Imaging Fudan University Shanghai China; ^2^ Department of Cardiology Zhongshan Hospital Shanghai Institute of Cardiovascular Disease Fudan University Shanghai China

**Keywords:** autophagosomes, codon, terminator, Danon disease, glycogen storage disease type IIb, LAMP2, whole exome sequencing

## Abstract

**Background:**

Danon disease is an X‐linked dominant hereditary condition caused by mutations in the gene encoding lysosomal‐associated membrane protein 2 (LAMP2), leading to failure of lysosome binding to autophagosomes, accumulation of glycogen in the heart, and abnormal cardiac function.

**Methods:**

We describe identification of a mutation in *LAMP2*, c.741+1G>T, in a family with Danon disease by whole exome sequencing.

**Results:**

Pathology examination of patient skeletal muscle biopsy showed myogenic damage and autophagic vacuoles with sarcolemmal features (AVSF). Numerous autophagic vacuoles accumulated in muscle cells were detected by electron microscopy, indicating abnormal autophagy function.

**Conclusion:**

The mutation did not result in loss of mRNA exons; rather, a 6‐nucleotide (two‐codon) insertion, where the latter was a stop codon, leading to early termination of LAMP2 protein translation. The resulting truncated protein lacks an important transmembrane domain, which will impair lysosome/autophagosome fusion, damage autophagy function, and result in the clinical manifestations of Danon disease.

Danon disease (OMIM #300257) is an X‐linked dominant genetic condition caused by mutations in the lysosomal‐associated membrane protein 2 (*LAMP2*) gene leading to a lysosomal disorder (Arad et al., 2005). The main clinical manifestations of Danon disease are cardiomyopathy, myopathy, and intellectual disability (D'Souza et al., 2014). Sugie et al. showed that all of 38 patients with Danon disease diagnosed by genetic analysis had cardiomyopathy, 84% had cardiac hypertrophy, and that 70% of male and 6% of female patients had intellectual disability (Sugie et al., 2002). Male patients often develop symptoms before 20 years of age, while most female patients with the condition develop symptoms in adulthood (Arad et al., 2005). Danon disease is characterized by hypertrophic cardiomyopathy of adolescent male onset, with elevated creatine kinase, skeletal muscle weakness, and intellectual disability. Symptoms are not prominent and are frequently overlooked or misdiagnosed as simple hypertrophic cardiomyopathy. Heart failure is an important factor affecting the prognosis of patients with Danon disease (Konrad et al., 2017). Pathological changes in the muscles of patients with Danon disease include the presence of multiple membranous autophagy vacuoles (Stokes, Taylor, McLean, D'Arcy, & Mariani, 2016).

The proband was an 18‐year‐old male. At the age of 3 years, the patient was diagnosed with cardiomyopathy and his physical development was somewhat delayed from an early age (Figure 1a). His intelligence is average, and he can complete everyday tasks and simple calculations. One year ago, the patient began to experience chest tightness after repeated activities, with dizziness, shortness of breath, and fatigue. He could achieve relief by taking a break. There has been a recent increase in admission of patients with chest tightness. Biochemical examination on admission indicated: alanine aminotransferase (ALT), 316 U/L (normal range, 9–50 U/L); aspartate aminotransferase (AST), 481 U/L (15–40 U/L); creatine kinase (CK), 3778 U/L (38–174 U/L); CK‐MB, 104 U/L; CK‐MM, 3,674 U/L; cardiac troponin, 0.26 ng/ml (0–0.03 ng/ml); N‐terminal pro‐b‐type natriuretic peptide, 2,154 pg/ml (0–300 pg/ml). Electrocardiogram revealed frequent atrial premature beats, ventricular pre‐excitation, complete left bundle branch block, and double ventricular hypertrophy. Echocardiography showed an increase in the left ventricle, thickening of the left and right ventricular walls, and abnormal myocardial echo, with reduced overall systolic activity (left ventricular ejection fraction [LVEF], 30%; tricuspid annular plane systolic excursion [TAPSE], 15 mm).

**Figure 1:**
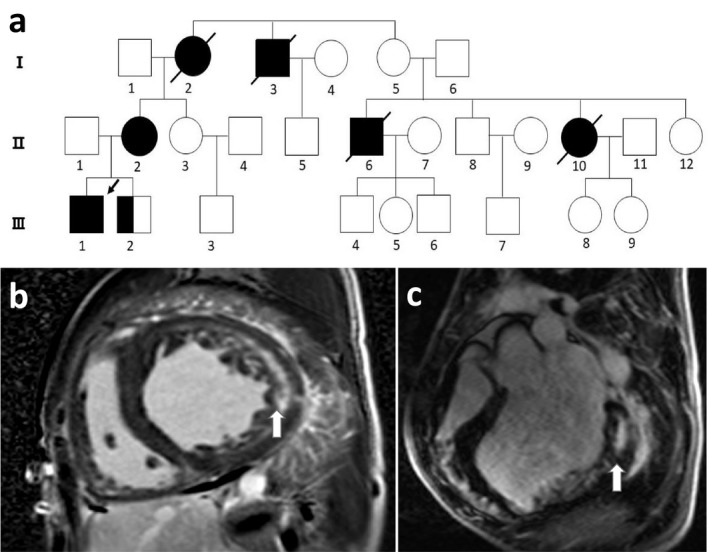
(a) Pedigree of the family included in this report. Squares, male individuals; circles, female individuals; slashes, deceased individuals; filled black shapes, affected patients; arrow, proband. (b, c) Cardiac MRI showing left and right ventricular wall hypertrophy, with reduced overall systolic activity, left atrioventricular enlargement, decreased myocardial T1 value, and delayed enhancement

The mother of the proband (II‐2) had a slightly enlarged left atrium and left ventricle, with no obvious abnormalities in left ventricular systolic function. The electrocardiogram waveform indicated atrial fibrillation. The patient's younger brother (III‐2; 13 years old) showed an ST‐T change in electrocardiogram and ventricular pre‐excitation. Echocardiography revealed a non‐uniform myocardial echo and normal contractile activity. Blood analysis showed liver and muscle enzyme abnormalities, CK, 2,217 U/L; lactate dehydrogenase, 1,155 U/L (109–245 U/L); ALT, 307 U/L; and AST, 377 U/L. There was no obvious abnormality in the limb muscles, nor was there atrophy of the skeletal muscle. The patient's father (II‐1) had normal electrocardiogram and echocardiography (Figure 2).

**Figure 2:**
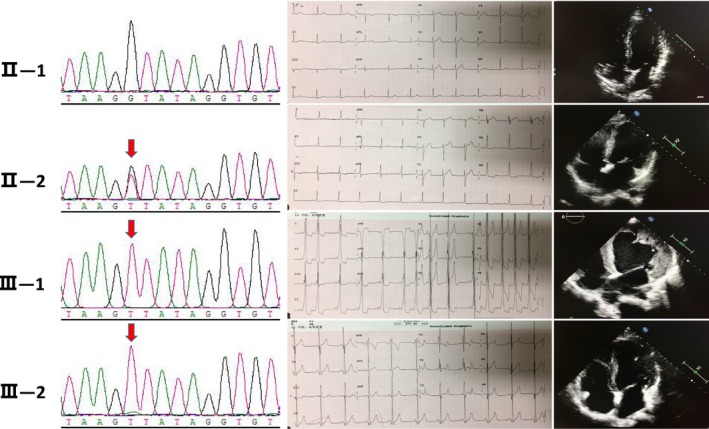
Sanger sequencing verifying that the proband (III‐1) and his mother (II‐2) and younger brother (III‐2) carried the mutation in *LAMP2*, c.741+1G>T. The patient's father (II‐1) did not carry the mutation. Electrocardiogram of the patients. Frequent premature atrial beats, ventricular pre‐excitation, and complete left bundle branch block were observed. Echocardiography showed enlargement of the left ventricle, thickening of the left and right ventricular walls, and abnormal echogenicity of the myocardium, with reduced myocardial contractile activity (LVEF, 30%; TAPSE, 15 mm). The electrocardiogram of the mother (II‐2) showed a waveform indicating atrial fibrillation. Echocardiography showed a slight increase in the left atrium and left ventricle, and left ventricular systolic function was acceptable. The electrocardiogram of the patient's brother (III‐2) showed ST‐T changes and ventricular pre‐excitation. Echocardiography showed non‐uniform echogenicity. The patient's father's (II‐1) electrocardiogram and echocardiogram were normal

Cardiac magnetic resonance imaging (MRI) of the patient revealed left and right ventricular wall hypertrophy, accompanied by a decrease in overall contractile activity, left atrioventricular enlargement, and decreased myocardial T1 value (Figure 1b,c). MRI is an important tool to assist in the identification of cardiomyopathy. Myocardial tissue composition and pathological changes are indirectly determined by measuring the longitudinal relaxation time (T1 time) of the myocardium. Most diseases, such as myocardial infarction, hypertrophic cardiomyopathy, and dilated cardiomyopathy, result in an increase in myocardial initial T1 (Stokes et al., 2016). Diseases with reduced initial T1 values are relatively rare and include metabolic cardiomyopathies, such as hemochromatosis, Fabry disease, and Danon disease; hence, this feature distinguishes these diseases from classical hypertrophic cardiomyopathy. In this patient, the T1 value of the heart was reduced, strongly indicating metabolic cardiomyopathy. Simultaneously, the patient's electromyographic report exhibited electrophysiological manifestations of myogenic damage, involving the limb muscles, and was active.

Given the findings from the examination of the index patient and his family, we considered that the disease was likely to be caused by a familial genetic mutation; therefore, we conducted genetic testing of the family and collect detailed clinical data from all family members. All subjects provided signed informed consent. Peripheral blood samples were collected from the subjects. The study was approved by the Ethics Committee of Zhongshan Hospital, affiliated to Fudan University (NO.2016‐16B).

Peripheral blood genomic DNA was extracted from blood samples using a TruSeq DNA Sample Preparation kit. DNA was sheared by sonication and ligated to sequencing adaptors to construct a library. The entire exome was captured using the Roche Nimblegen SeqCap EZ v5.1 kit for high‐throughput sequencing on the Illumina XTEN platform. The resulting data quality Q30 ratio was >90%, the median sequencing depth across the exon target region was >50×, the sequencing depth of 95% of the target region was >30×, and the exon loss rate was <0.2%. Raw genome sequence data were analyzed by comparison using the bwamem/disco vardenovo/freebayes process to extract single nucleotide variant (SNV) and structural variant (SV) data. Copy number variant (CNV) data were evaluated using EulerCNV and CNVnator. SNV data were annotated using data from our in‐house cohort, dbSNP138, and 1000G_OMNI2.5, and the annotated data analyzed using snpEff and deep neural networks developed in‐house. Mutation locus prediction was conducted using an in‐house C++/R deep neural network model workflow. CNV/SV data were filtered by comparison with in‐house cohort data, along with 1000G and public CNV database data, and are reported using Langya. Signal transduction pathway analysis was performed using an in‐house R/Python/Perl package. OMIM/Monarch/ExAC database analysis was conducted using an in‐house Perl/R application program interface. Neural network analysis of phenotype–genotype relationships was performed using Langya/Marshes. Data interpretation was conducted in accordance with the relevant guidelines of the American College of Medical Genetics and Genomics (ACMG; Zhou et al., 2018). The variation was named according to Human Genome Variation Society (HGVS) recommendations (http://www.hgvs.org/mutnomen/).

By whole exome sequencing analysis, we identified a novel pathogenic mutation in *LAMP2* c.741+1G>T. Sanger sequencing confirmed that the proband, his mother, and his brother are carriers of this mutation. *LAMP2* has 9 exons and encodes the LAMP2 protein, which consists of 410 amino acids, comprising three domains: the lysosomal, transmembrane, and cytoplasmic regions. Exons 1–8 of *LAMP2* encode the intra‐lysosomal domain, while the ninth exon encodes the transmembrane region and the cytoplasmic domain. We took a muscle biopsy from the patient, extracted RNA, reverse transcribed it to generate cDNA, and conducted PCR and sequencing analysis. The results indicated that the identified mutation resulted in the insertion of 6 nucleotides in the mRNA, with the latter three bases forming a stop codon, which resulted in early termination of protein translation and loss of the LAMP2 transmembrane domain (Figure 3). Hence, in this case, the *LAMP2* mutation did not cause exon loss in the mRNA, rather the insertion of six additional nucleotides, the second of which happened to be a stop codon. This alteration led to complete loss of the protein encoded by the transcript after exon 5, including the crucial transmembrane domain. This change will affect the fusion of lysosomes and autophagosomes, and impair the autophagy function of cells, resulting in clinical manifestations.

**Figure 3:**
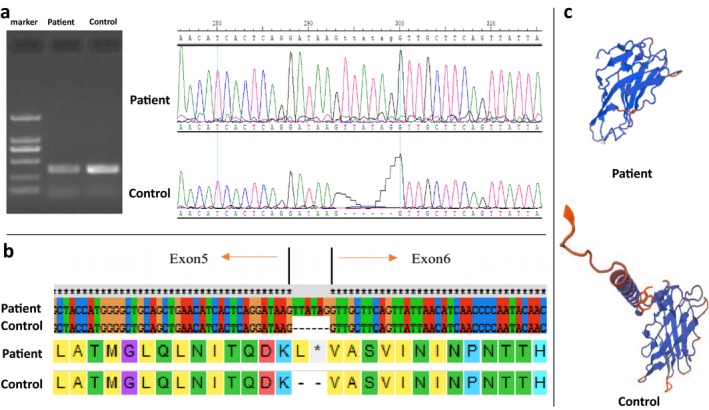
RNA was extracted from a patient muscle biopsy specimen and reverse transcribed into cDNA. After PCR, sequencing revealed that the mutation resulted in the insertion of 6 nucleotides in the mRNA (a). This is predicted to result in the early termination of the translated protein (b), ultimately leads to the loss of the LAMP2 protein transmembrane domain (c)

We took a biopsy from the right bicep of the patient. The process of muscle tissue fixation is as described previously (Cetin et al., 2016). Pathological analysis revealed numerous vacuoles in the myocytes, positive periodic acid‐Schiff (PAS) staining in the vacuoles, and positive staining of the vacuolar edge membrane protein, indicating myogenic damage and autophagic vacuoles with sarcolemmal features (AVSF). Further electron microscopy evaluation showed that the myofilaments were basically normal, with large numbers of autophagosomes, lipid vesicles, and myeloid bodies accumulated in the perinuclear region. No mitochondrial abnormalities were identified, while the sarcoplasmic reticulum was slightly dilated, glycogen was increased, and local myofibrils were absent (Figure 4). Finally, we diagnosed the patients with Danon disease, based on their medical history, genetic test results, and family history.

**Figure 4:**
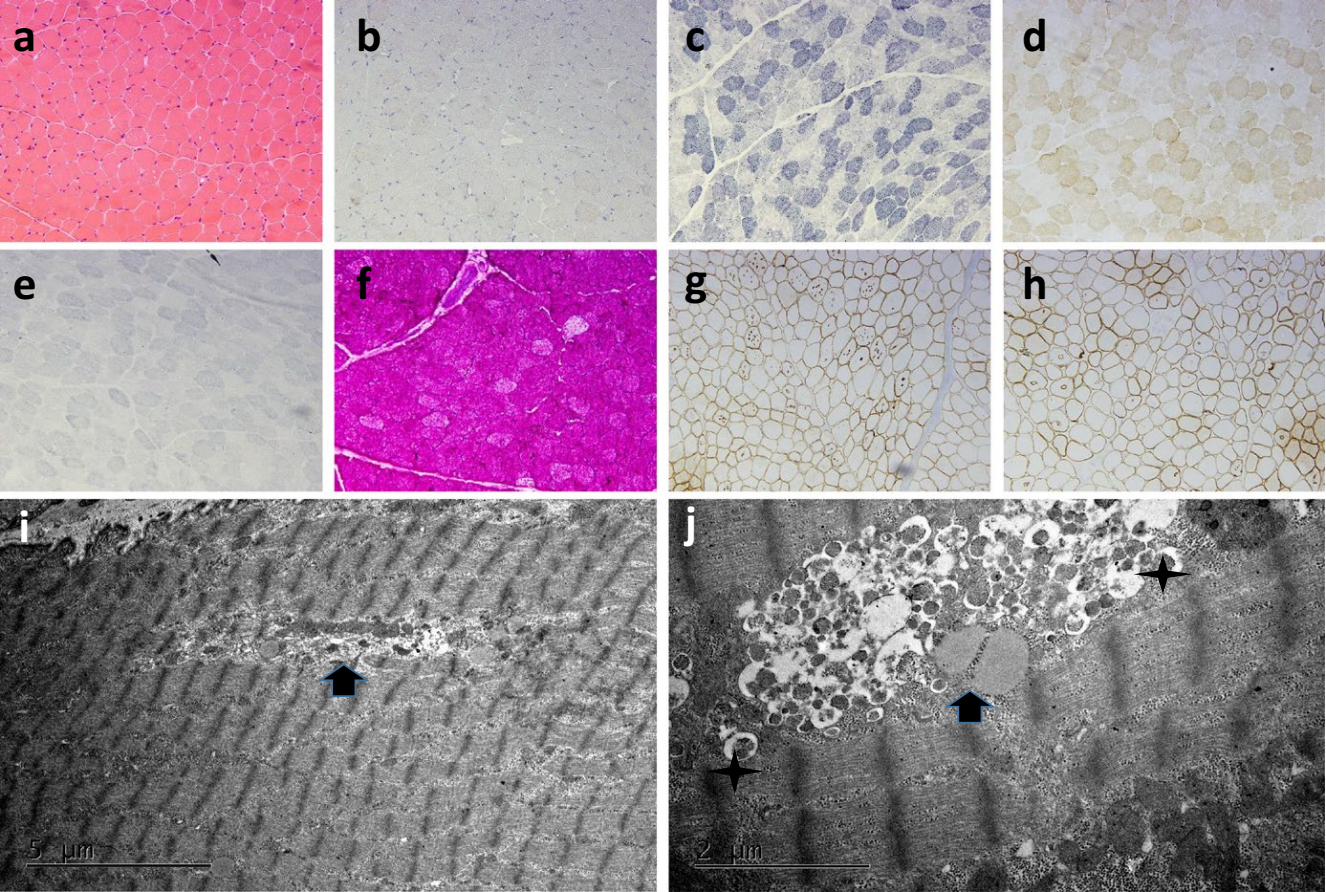
Muscle biopsy pathology staining (a:HE, b:COX, c:NADH, d:SDH, e:PAS, f:ORO, g: rod‐dystrophin, h: α‐sarcoglycan). There was no obvious fibrous tissue hyperplasia in the fascia and mild hyperplasia of the endomysium. No obvious inflammatory cell infiltration was observed. Muscle cells differed slightly in size, and morphology was regular. A large number of muscle fiber vacuoles were observed. No obvious necrosis or new muscle fibers were observed, and there was no obvious muscle atrophy of the perivascular muscle fibers. Modified Gomori‐trichrome staining did not reveal ragged red fibers. The structure of the NADH myofibril network was generally normal. COX staining did not reveal COX‐negative fibers. Some muscle fiber enzyme activities were slightly non‐uniform. Succinate dehydrogenase staining did not reveal RBF or SSV. Muscle tissue was deeply stained with periodic acid‐Schiff (PAS); PAS‐positive substances were deposited in the vacuoles, and PAS stained the muscle fibers. R; N; C‐Dys (+); α; β; γ‐Sar (+); Dysf (+); MHC‐I (−), positive vacuolar edge membrane protein staining. Electron microscopy. (i,j) Examination of myofilaments indicated that they were basically normal. There were a large number of autophagosomes, lipid vesicles, and myeloid deposits in the perinuclear region

Metabolic cardiomyopathy includes Fabry disease, Danon disease, Pompe disease, and Protein Kinase AMP‐Activated Non‐Catalytic Subunit Gamma 2 (PRKAG2) cardiac syndrome. These conditions all cause cardiac hypertrophy resulting from mutations in non‐sarcomeric genes (Yardeni et al., 2017). The glycogen accumulation disease caused by *LAMP2* mutations is an X‐linked condition, characterized by cardiomyopathy, skeletal myopathy, and varying degrees of intellectual disability (Chen et al., 2012). For patients with *LAMP2* mutations, the age of onset is earlier than that observed in other types of metabolic disease, and it occurs earlier and more frequently in men (Di Blasi, Jarre, Blasevich, Dassi, & Mora, 2008; Nadeau, Therrien, Karpati, & Sinnreich, 2008). Simultaneously, most patients have elevated serum ALT and CK. The condition is often manifested as hypertrophic cardiomyopathy, usually accompanied by pre‐excitation syndrome.

To date, there is no specific treatment for Danon disease. Malignant ventricular arrhythmias and heart failure are the leading causes of death in patients with this condition (Cetin et al., 2016; Cheng et al., 2012). Cardiac MRI is very sensitive to changes in myocardial fibrosis and can assist in identifying patients at high risk of future arrhythmia events. The most effective clinical treatment for Danon disease is heart transplant, which can significantly improve patient survival (Sugie et al., 2016). As the disease usually progresses rapidly, it is recommended cardiac function is assessed in male patients every 3–6 months, including assessment of cardiac transplantation. Genetic counseling should also be provided for patients with Danon disease and their relatives, to reduce the risk in future generations (Bottillo et al., 2016). As the pathogenesis of Danon disease is gradually elucidated, and gene therapy and cell transplantation techniques are developed, the future may bring effective treatments for genetic diseases, including Danon disease.

## CONFLICT OF INTERESTS

The authors have no conflict of interest to declare.
